# Evaluating the Experience of Teen-to-Teen Crisis Line Volunteers: A Pilot Study

**DOI:** 10.1007/s10597-024-01298-z

**Published:** 2024-06-04

**Authors:** Catherine R. Glenn, Taylor Kalgren, Sandipan Dutta, Raksha Kandlur, Kelsie K. Allison, Annie Duan, Cheryl Karp Eskin, Morgan Leets, Madelyn S. Gould

**Affiliations:** 1 Old Dominion University, Norfolk, VA USA; 2 Virginia Consortium Program in Clinical Psychology, Norfolk, VA USA; 3 University of Rochester, Rochester, NY USA; 4 Teen Line, Los Angeles, CA USA; 5 YouthLine, Portland, OR USA; 6 Columbia University, New York City, NY USA

**Keywords:** Adolescents, Crisis line, Peer support, Suicide prevention

## Abstract

Teen-to-teen (t2t) crisis lines are a special type of crisis service where youth volunteers help their peers. Although prior research has examined the experience of adult crisis line responders, no research has examined the experience of adolescents who do this work. In collaboration with two of the largest t2t lines in the U.S., this pilot study is the first examination of t2t crisis line work. Volunteers (ages 14–20) reported: their primary motivation for joining the crisis lines was to *help others and give back to the community*; responding to a range of peers’ problems on the t2t crisis line, including high-risk suicide contacts; and a range of ways the crisis line work impacted their lives. Findings provide preliminary information about the experience of adolescents engaging in t2t crisis line work. Additional research is needed in larger and more diverse samples to understand the impact of crisis line work for youth.

**Supplementary Information:**

The online version contains supplementary material available at 10.1007/s10597-024-01298-z.

## Introduction

The U.S. is experiencing an unprecedented mental health crisis among youth. Suicide is a leading cause of death among adolescents in the U.S., and, alarmingly, youth suicide rates have continued to rise over the past 10 years (Centers for Disease Control and Prevention, 2018). Non-fatal suicidal thoughts and behaviors are even more common. The most recent Youth Risk Behavior Survey in the U.S. indicates that 22% of high school students reported thinking about suicide in the past year and 10% attempted suicide at least once (Centers for Disease Control and Prevention, 2019). High rates of suicidal thoughts and behaviors are concerning as they cause significant impairment in academic and social domains (Copeland et al., 2017; Foley et al., 2006), and increase risk for suicide death (Ribeiro et al., 2016).

### Crisis Lines

Effective suicide prevention requires a comprehensive approach integrating a range of upstream (e.g., enhancing life skills and resilience) and downstream strategies (e.g., responding to a crisis; JED Foundation, 2017; Suicide Prevention Resource Center, 2020). Crisis hotlines are one of the oldest downstream approaches for suicide prevention (Litman et al., 1965; Shneidman & Farberow, 1956). Since the 1950s, crisis hotlines have been providing critical support and mental health resources to callers during mental health emergencies, including suicidal crises. As such, crisis hotlines have been viewed as a national “safety net” in the landscape of suicide prevention.

Decades of research on crisis hotline effectiveness was essential in advocating for, and supporting, the 2022 passage of the U.S.’s National Suicide Hotline Designation Act, which established a new three-digit number (988) for a national suicide prevention and mental health crisis hotline. For instance, crisis line contacts have been found to improve adults’ depression (Gould et al., 2022; Kalafat et al., 2007; Mishara & Daigle, 1997), emotional distress (Coveney et al., 2012; Gould et al., 2021; Kalafat et al., 2007; Ramchand et al., 2017; Shaw & Chiang, 2019), hopelessness (Coveney et al., 2012; Gould et al., 2007, 2022; Kalafat et al., 2007; Mishara et al., 2007), and suicide risk (i.e., thoughts, plans, intent; Coveney et al., 2012; Gould et al., 2007, 2016, 2021, 2022; Mishara & Daigle, 1997; Shaw & Chiang, 2019).

Notably, as texting and online messaging (e.g., chat) have become the dominant forms of communication for younger people (Lenhart et al., 2015), crisis hotlines worldwide have expanded from the traditional hotline format (i.e., phone calls) to include text and chat features (Evans et al., 2013; Fukkink & Hermanns, 2009; Gibson et al., 2016; Haxell, 2015; Mokkenstorm et al., 2017; Sindahl et al., 2019; Thompson et al., 2018). Youth report a preference for texts/chats over phone calls because of the ease, privacy, and confidentiality provided (Evans et al., 2013; Gibson et al., 2016; Haxell, 2015; Nesmith, 2018), and report being more likely to share mental health information via these methods (Evans et al., 2013; Nesmith, 2018). Youth also report preferring to text with “strangers” because they are perceived as less judgmental than friends (Evans et al., 2013). For these reasons, crisis line text/chat services may help overcome stigma related to help-seeking—a major barrier to care for youth (Gulliver et al., 2010; Sylwestrzak et al., 2015). Although less research has examined the effectiveness of crisis lines for youth, studies to date have been promising and suggest that youth report: less distress and depression (Gould et al., 2021), lower suicide risk (Gould et al., 2021; Sindahl et al., 2019), and greater well-being following crisis line contacts (Fukkink & Hermanns, 2009), as well as satisfaction with and helpfulness of these resources (Gould et al., 2022; Nesmith, 2018).

### Crisis Line Responders

The majority of crisis line research has examined the effectiveness for people who use this resource. Given that the effectiveness of crisis lines relies on the responders (who answer the calls, texts, and chats), it also is critically important to understand the responders’ experience providing this service. Most crisis hotlines are staffed by adults (paid employees or volunteers). Adults report a range of motivations for pursuing crisis hotline work, including (1) self-directed reasons such as gaining new skills and experiences (Mishara & Giroux, 1993; Praetorius & Machtmes, 2005; Sundram et al., 2018), and increasing self-knowledge (Praetorius & Machtmes, 2005; Rath, 2008), and (2) other-directed reasons such as helping others (Hector & Aguirre, 2009; Mishara & Giroux, 1993; Praetorius & Machtmes, 2005; Sundram et al., 2018) and meeting people (Mishara & Giroux, 1993).

Beyond motivations for pursuing crisis hotline work, some research has explored the impact of the crisis line experience on adult staff. Findings have been mixed (Kitchingman et al., 2018b; Willems et al., 2020) with most research examining negative outcomes (Kitchingman et al., 2018b; Willems et al., 2020). Although integration of existing research is challenging, due to varying study designs and methods, some studies have found that crisis line work leads to increased psychological distress (Dunkley & Whelan, 2006; Kitchingman et al., 2017, 2018a). In addition, some studies (Cyr & Dowrick, 1991), but not all (Roche & Ogden, 2017), have observed increased burnout (i.e., physical and/or emotional exhaustion resulting from work-related stress). However, negative effects have not been observed across all domains. For instance, across several studies, adult crisis line volunteers have *not* reported increased secondary traumatic stress (Dunkley & Whelan, 2006; Furlonger & Taylor, 2013; Kitchingman et al., 2018a). Moreover, two studies found no significant increases in suicide ideation among adult crisis line workers (Kitchingman et al., 2017, 2018a). Although limited research has specifically examined the positive impact of crisis line work for adults (rather than just the absence of negative outcomes; Willems et al., 2020), one study found that crisis line volunteers reported high satisfaction and intention to stay with the crisis line (Hellman & House, 2006). In summary, prior research indicates that crisis line work may have some positive as well as some negative impacts on adults. However, no research to date has examined the impact of crisis line work for *youth* volunteers working on teen-to-teen crisis lines.

### Teen-to-Teen Crisis Lines

Teen-to-teen (t2t) crisis lines are a specific type of crisis service in which youth volunteers help their similarly aged peers (through texts and chats [most common], phone calls, and emails). Specifically, youth volunteers (starting at 14 years-old) staff crisis lines during specified evening hours to provide their peers with support and resources related to a variety of topics, including high-risk safety concerns (e.g., suicide risk and child maltreatment). Before starting on the t2t crisis line, youth volunteers complete extensive (60 + hours) training in crisis intervention skills, t2t crisis line protocols, and multicultural awareness. Once on the line, youth are closely supervised by clinically trained adult staff.

T2t crisis lines may be a promising resource given the important role peers can play in suicide prevention (Ali et al., 2015; Katz et al., 2013; Robinson et al., 2013). Decades of research have found that youth are more likely to disclose their suicide risk to peers than to adults (Kalafat & Elias, 1992; Michelmore & Hindley, 2012; Rickwood et al., 2005; Ross, 1980; Wyman et al., 2008). For instance, peers play a key role in school-based prevention programs (e.g., gatekeeper trainings; Katz et al., 2013; Robinson et al., 2013) and online supports for mental health (e.g., chat rooms; Ali et al., 2015). Moreover, peer support models have demonstrated utility across the lifespan for a range of physical and mental health conditions (Davidson & Guy, 2012; Haines et al., 2018; Pfeiffer et al., 2011; Tomasino et al., 2017).

T2t lines have the potential to offer a unique support experience both for the youth who use the lines and for the youth volunteers who respond to the contacts (Croton et al., 2020). Youth volunteers may benefit from this t2t experience (e.g., helping peers, sense of community, and skill development), but there is also potential for negative or harmful experiences as observed in some studies with adults (Kitchingman et al., 2018b; Willems et al., 2020). It is essential to examine the impact on youth volunteers specifically as young people may be particularly vulnerable to the stress and potential traumatization from crisis line work (Insel & Gould, 2008), and some concerns have been raised about the impact of these t2t lines for youth (Lewis & Lewis, 1996). However, to date, research has not yet examined the experience of youth volunteers who staff crisis lines.

### Current Study

The current longitudinal pilot study is the first to examine the experience of t2t crisis line work for youth volunteers in collaboration with two of the largest t2t lines in the U.S.—Teen Line and YouthLine. Although the sample is small, this study is an important step in exploring what it is like for teens to engage in this type of intensive crisis line work. Specifically, this study examined: (1) youth volunteers’ motivations for pursuing crisis line work, (2) content of crisis contacts volunteers responded to, (3) aspects of the positive experience of t2t crisis line volunteerism, and (4) aspects of the negative experience of t2t crisis line volunteerism for youth. Given that prior research in this area has focused on adult populations, our analyses were considered exploratory, and no specific hypotheses were made.

## Method

### Participants

Twenty teen-to-teen crisis line volunteers, 14–20 years old (*M* = 16.95, *SD* = 1.15), were enrolled in the current pilot study (see Table 1): six (30%) were from Teen Line and 14 (70%) were from YouthLine (see *Procedure*). At baseline, volunteers’ length of experience on the crisis line varied: 20% had been volunteers for 1–2 months, 20% for 3–6 months, 5% for 7–11 months, 50% for 1–2 years, and 5% for more than 2 years. Eleven volunteers (55%) reported lived experience with suicidal thoughts and behaviors before joining the crisis line (see *Measures*).

**Table 1:** Teen-to-teen crisis line volunteer demographics (at study enrollment).^1^

Demographic variable	*N* = 20
Age (years): *M (SD)*	16.95 (1.15)
Grade: *%*	
9th	5%
10th	10%
11th	40%
12th	35%
College	10%
Gender identity^2^: *%*	
Cisgender female	90%
Cisgender male	5%
Not sure	5%
Sexual orientation^3^: *%*	
Heterosexual/straight	50%
Bisexual	25%
Lesbian/gay	15%
Not sure	10%
Race^4^: *%*	
White or Caucasian	85%
Multiracial or other self-identified race	10%
Asian	5%
Ethnicity: *%*	
Not Hispanic or Latinx/e	95%
Hispanic or Latinx/e	5%
Currently living with: *% (Selected all that apply)*	
Mother (biological/adoptive/step/foster)	95%
Father (biological/adoptive/step/foster)	85%
Siblings (biological/adoptive/step/foster)	60%
Extended family	5%
Family friends	5%
Friend/roommate	5%
Boyfriend, girlfriend, romantic partner	5%

^1^ Given the small sample size, volunteer demographics are combined across the two sites

^2^ The following categories for gender identity were provided but not endorsed: transgender, non-binary, agender, genderfluid, and prefer to self-describe

^3^ The following categories for sexual orientation were provided but not endorsed: pansexual, asexual, queer, and prefer to self-describe

^4^ The following categories for racial groups were provided but not endorsed: American Indian or Alaska Native, Black or African American, and Native Hawaiian or Pacific Islander

### Procedure

The study was conducted in accordance with the Declaration of Helsinki, and the following procedures were approved by the Institutional Review Board at Old Dominion University.

#### Volunteer Recruitment and Consent

T2t crisis line volunteers, ages 14–20, were recruited from two of the largest teen-to-teen crisis lines in the U.S.—Teen Line and YouthLine. Volunteers were recruited through informational flyers and emails distributed by the t2t line staff. Parents (for volunteers 14–17 yo.) were provided with information about the study by email. Either adolescent assent and parent permission (for 14–17 yo.) or adolescent consent (for 18–20 yo.) was obtained prior to study initiation. Participation in this study was voluntary and not disclosed to Teen Line/YouthLine staff, except for cases of high or imminent suicide risk (see *Risk and Safety monitoring* section).

#### Teen Line

Teen Line (est. 1980) is a nonprofit, community-based organization in Los Angeles, CA that operates one of the major t2t crisis lines in the U.S.^1^ Teen Line offers a range of contact options for youth, including text (most common), phone calls, and emails. Youth volunteers (14–18 yo.) are available on the hotline four hours per day (6-10pm PST). In 2019, Teen Line received 21,157 contacts (texts, calls, and emails) from across the U.S. Approximately 150 youth volunteers work at Teen Line at a given time. Based on the training required, LA-area youth are eligible to join Teen Line from the fall of 9th grade to the summer after 10th grade. A 1-year commitment is required, and volunteers typically participate for 2–3 years. Selection of volunteers is based on an application and interview process assessing peer helper interest, social skills, coping skills, insight, maturity, support system, and lived experience related to mental health problems. Teen Line encourages youth with lived experience to volunteer, but is careful to assess their recovery, coping skills, and support system. Didactic training is 65 h over 12 weeks, including active listening, foundational crisis assessment and intervention skills, and multicultural awareness and competency. Next, trainees complete a series of “observer” tasks (e.g., observe existing volunteers and complete a series of roleplays) before they transition to becoming independent volunteers. The training process takes 6–12 months to complete. Once eligible, volunteers must complete two shifts per month, and most complete weekly shifts (each 4.5 h long). During shifts, volunteers are closely supervised by master’s level clinicians. Supervisors debrief with volunteers after high-risk and difficult calls, and also “check out” at the end of each shift (i.e., discuss how the shift went, and if they had any high-risk or difficult calls, before heading home). Moreover, supervisors discuss any concerns for their volunteers and hotline issues during weekly meetings. During the recruitment period for this pilot study, the overall demographics of Teen Line volunteers included: Age: average 15 years old; Gender: 79% cisgender female, 16% cisgender male, 5% transgender or gender diverse; Race: 57% White, 21% Multiracial, 9% Asian, 6% Black; and Ethnicity: 7% Hispanic/Latine.

#### YouthLine

YouthLine (est. 2000) is a crisis and t2t support service for adolescents based in Portland, OR. The service is provided by *Lines for Life* (est. 1993), which is a non-profit organization dedicated to preventing substance abuse and suicide. YouthLine offers a range of contact options for youth, including text (most common), chat, phone calls, and emails. Youth volunteers (15–20 years old) are available on the hotline six hours per day (4-10pm PST). In 2020, YouthLine received 28,190 contacts (texts, calls, and emails) from across the U.S. Between 100 and 150 youth volunteers work at YouthLine at a given time. Youth are eligible to join YouthLine if they are local, meet the age requirement, and can make a 1-year commitment. Most volunteers participate for several years. Selection of volunteers is similar to Teen Line with the same lived experience considerations. Training is 65 + hours, including didactics (e.g., safeTALK, Youth Mental Health First Aid, plus skills noted for Teen Line training), roleplays, and shadow shifts. The training process takes 3–5 weeks to complete. Volunteers commit to at least weekly shifts (each 3.5–4.5 h long). During shifts, volunteers are closely supervised by master’s level clinicians and/or staff with extensive crisis work experience. Supervisors monitor all contacts and provide feedback as needed during and after the contact. Supervisors debrief with volunteers after high-risk and difficult calls, and “check out” at the end of shifts (i.e., wrap up their shift, debrief, and plan for self-care). In addition, volunteers have access to a “volunteer text line” for support. During the recruitment period for this pilot study, the overall demographics of YouthLine volunteers included: Age: average 16 years old; Gender: 79% cisgender female, 10% cisgender male, 2% transgender or gender diverse (remaining % missing); Race: 70% identified as White only, 24% identified as a person of color, and 6% preferred not to answer; with respect to the racial groups (volunteers could select all that apply), 77% White, 22% Asian (5% East Asian, 15% South Asian, 2% Southeast Asian), 2% Black, 2% Middle Eastern or North African; and Ethnicity: 8% Hispanic/Latine.

#### Data Collection

Volunteers were asked to complete a 20-minute survey up to five times during their volunteer position—baseline and then every three months (approximately every 12 shifts as most volunteers complete a weekly shift) for up to one year (i.e., baseline, 3 months, 6 months, 9 months, and 12 months). Surveys were completed online (via Qualtrics) ideally at the beginning of a volunteer shift for risk and safety monitoring (see next section). For this pilot study, participants volunteered their time, and were provided information about mental health resources after each survey. Data for this longitudinal pilot study were collected from August 2020 to May 2021 for the baseline, and until May 2022 for the 12-month follow-up. Retention rates over the 1-year follow-up were: Time 2 (85%), T3 (75%), T4 (70%), and T5 (55%). Reasons for attrition included: (a) ending volunteer position at the crisis line (*n* = 7; e.g., leaving the area to go to college), and (b) active withdrawal/opt out of study or lost to follow-up, with no reason provided (*n* = 2).

#### Risk and Safety Monitoring

To ensure volunteers’ safety during the study, the research team monitored volunteers’ surveys, specifically responses that would indicate high or imminent risk for suicide (e.g., indicating suicidal thoughts, plans, or attempts within the past 30 days). Concerns about volunteers’ suicide risk were reported to the t2t line supervisor to reach out to the volunteer as needed. This risk and safety monitoring protocol was developed in collaboration with the t2t crisis lines. Adolescent volunteers and parents were informed about this aspect of the protocol during the consent process (for volunteers, confidentiality was reviewed verbally [over the phone], in addition to the written assent/consent form).

### Measures and Data Analysis

#### Background Information

Volunteer demographics (age, grade, gender identity, sexual orientation, race, ethnicity, and current members living in their home) were assessed at baseline. In addition, suicidal thoughts and behaviors (STBs) were measured over volunteers’ lifetime (indicating lived experience) with the CDC’s Youth Risk Behavior Survey (YRBS) STB questions, which have been widely used to assess STBs in youth (Centers for Disease Control and Prevention, 2019; May & Klonsky, 2011; Pinzon-Perez & Pérez, 2001; Shilubane et al., 2013).

#### Motivations for Joining the t2t Line

Volunteers’ motivations for joining the crisis line were assessed during the baseline survey using one open-ended question: *“Why did you join YouthLine/Teen Line?”* Responses were examined using qualitative coding procedures, including directed content analysis and thematic analysis (Hsieh & Shannon, 2005). Specifically, a codebook (categories, definitions, and examples) was developed by a first (graduate-level) coder [co-author TK], with supervision from a senior coder [co-author CG], based on prior research with adults and input from the t2t crisis line collaborators (see Supplement 1). The codebook was applied to the data. If free response data did not fit into an existing category, a new, mutually exclusive category was created and added to the codebook. This process was repeated until the point of saturation (i.e., when the codebook was sufficient to code all free response data, and no new themes were identified). Once complete, the codebook was passed to a second, independent (graduate-level) coder [co-author KA]. Discrepancies between coders were resolved with a third, senior coder [co-author CG].

#### Content of Crisis Line Contacts

The content of crisis contacts (across calls, texts, chats, emails) volunteers responded to were assessed in each survey through a series of checkboxes (see Table 2). Endorsement of content categories were examined within person across all surveys (i.e., each volunteer counted once). The content of the contacts was assessed, but not the specific mode of communication (e.g., text, call, etc.).

**Table 2:** Types of content responded to by volunteers on the teen-to-teen crisis lines

Content types (calls, texts, chats, emails)	*N* = 20 (%)^1^
Depression, anxiety, or other mental health symptoms	100%
Relationship difficulties (friend, family, romantic partner, etc.)	100%
Abuse, assault, or violence *(not currently high risk)*	95%
Academic stress	95%
Bullying/harassment	95%
COVID-19 or quarantine stress	95%
Self-injury or suicide *(not currently high risk)*	95%
**High-risk** abuse, assault, or violence (i.e., current/ongoing abuse, recent assault, recent domestic violence)	90%
**High-risk** suicide and self-injury (i.e., actively self-harming or unable to move away from means to self-harm, suicidal thoughts with intent and/or a plan, recent suicide attempt)	90%
Stressors related to gender identity or sexual orientation	90%
Grief/mourning loss of loved one	85%
Negative self-image/body or weight concerns	85%
Pregnancy, STIs	85%
Substance abuse/addiction	65%
Financial concerns	60%
Racism, racial injustice, murders of Black men and women, protests related to these concerns	45%
*Other content areas reported by volunteers (provided in write-in option)*:	
Disabilities	10%
Loneliness/isolation	10%
Religious issues	10%
Struggles with aspects of identity *(not covered in categories above)*	10%
Lack of access to mental health resources	5%
Legal issues	5%
Runaway	5%
Stress about the future	5%

^1^ Content aggregated within person across all reported surveys (i.e., each person counted once)

#### Positive Impact of t2t Line Volunteerism

Given limited prior research on positive outcomes, the research team developed a series of brief quantitative and qualitative questions assessing: (1) volunteers’ overall positive/helpful experience on the t2t line (single item on a 5-point scale from 0 = *Not at all* to 4 = *Extremely positive*), (2) the specific domains found to be the most positive/helpful, based on prior research with adults and in collaboration with staff at the two t2t crisis lines (checkboxes and free response option; see Table 3a), and (3) how the t2t crisis line experience positively impacted their life outside of the volunteer work (checkboxes and free response option; see Table 4a). Endorsement of specific positive domains at the t2t crisis line and positive impact outside of the crisis line were examined, across all survey time points, as descriptive frequencies. “Other” (write-in) options were examined to determine whether they fit into an existing category, or a new category needed to be created. Responses to the one-item positive experience rating question were averaged across all surveys completed.

**Table 3:** Positive/helpful and negative/unhelpful aspects of working on the teen-to-teen crisis lines

3a. Positive/helpful aspects of working on the teen-to-teen crisis lines	*N* = 20 (%)^1^
Helping others	100%
Skills learned (e.g., knowing “what to do” in a crisis, improved communication skills)	100%
Work is a good match with my skills and values	100%
Being accepted by others (for who I am)	95%
Sense of belonging	95%
Provides me with a useful perspective on my own experiences	90%
Relationships/friendships (e.g., with other volunteers)	90%
Adults I can trust	80%
How to cope with my own problems	80%

3b. Negative/unhelpful aspects of working on the teen-to-teen crisis lines^2^	*N* = 20 (%)^1^
Stressful work	70%
Negative impact on my own mental health	40%
Minimizing my own problems (compared to those who use the crisis line)	25%
High expectations of volunteer position	20%
Too much of a time commitment	5%
*Other themes described by volunteers (provided in write-in option)*:	
Interpersonal stressors within the crisis line community	20%
Not feeling competent or efficacious	10%

^1^ Categories aggregated within person across all reported surveys (i.e., each person counted once)

^2^ Of note, 10% of volunteers did not report any negative experiences

**Table 4:** Positive and negative impact on life outside of the teen-to-teen crisis lines

4a. Positive impact on life outside of the teen-to-teen crisis lines	*N* = 20 (%)^1^
Greater empathy for others	100%
Improved listening and communication skills	100%
Better understanding of the issues teens face	95%
Changed the way I talk about or view mental health/illness	95%
Greater sense of purpose	95%
Greater confidence	90%
Helped me think through my future career plans	90%
Increased understanding of own boundaries and knowing when to get others’ help	90%
Relevant job experience and references for future employment	90%
Improved decision-making	85%
Increased own help-seeking or treatment-seeking	85%
Appreciation for my own life	80%
Improved relationships with friends and family	70%
Improved time management	60%

4b. Negative impact on life outside of the teen-to-teen crisis lines^2^	*N* = 20 (%)^1^
Increased pressure to support others’ mental health because of work on the crisis line	65%
Emotionally drained	60%
Increased stress or anxiety	40%
Less time for other activities outside of volunteer experience	20%
Negative impact on my own mental health	20%
Worsened relationships with friends and family	0%

^1^ Categories aggregated within person across all reported surveys (i.e., each person counted once)

^2^ Of note, 10% of volunteers did not report any negative experiences

#### Negative Impact of t2t Line Volunteerism

Like overall positive impact, the research team developed a series of brief quantitative and qualitative questions assessing: (1) volunteers’ negative/unhelpful experience on the t2t line (single item on a 5-point scale from 0 = *Not at all* to 4 = *Extremely negative*), (2) the specific domains found to be most negative/unhelpful, based on prior research with adults and in collaboration with staff at the two t2t crisis lines (checkboxes and free response option; see Table 3b), and (3) how the t2t crisis line experience negatively impacted their life outside of the volunteer work (checkboxes and free response option; see Table 4b). Endorsement of specific negative domains at the t2t crisis line and negative impact outside of crisis line were examined, across all survey time points, as descriptive frequencies. “Other” (write-in) options were examined to determine whether they fit into an existing category, or a new category needed to be created. Responses to the one-item negative experience question were averaged across all surveys completed.

Finally, several measures based on previous research were used to examine volunteers’ mental well-being. Secondary traumatic stress was measured with a 10-item subscale from the Professional Quality of Life Measure (ProQOL; Stamm, 2010)—a validated scale (Hemsworth et al., 2018; Heritage et al., 2018) used widely in prior research with human services workers, including crisis line workers, to assess components of compassion fatigue (Geoffrion et al., 2019; Louison Vang et al., 2020). Psychological distress was assessed with the brief (6-item) Kessler-6 (Kessler & Mroczek, 1992, 1994; Kessler et al., 2002) which measures recent anxiety and depression symptoms and has been validated with youth (Chan & Fung, 2014; Green et al., 2010; Mewton et al., 2016; Peiper et al., 2015). Given the small sample, secondary traumatic stress and psychological distress were averaged across all surveys completed (rather than examined over time).

## Results

### Motivations for Joining the t2t Crisis Line

The directed content analysis and thematic analysis of motivations for joining the t2t crisis line resulted in six major themes–three categories were identified prior to coding and three were new categories based on responses in this sample (see Supplement 1). The most common motivations for joining the crisis line were: to *help others and give back to the community* (95% of the sample endorsed), followed by *to gain new skills* (40%), and *given my own or close others lived experience with mental health issues* (35%). Three new categories were identified during coding: *to learn more about mental health [broadly]* (20%), *to destigmatize or raise awareness about mental health conditions* (15%), and *to respond to the demand for mental health services and need for equitable mental healthcare* (10%).

### Content of Crisis Line Contacts

The most frequent content of t2t crisis line contacts, reported by 100% of volunteers, were (1) depression, anxiety or other mental health symptoms, and (2) relationship difficulties (see Table 2). Notably, almost all volunteers (90%) reported *high-risk* contacts related to *suicide and self-injury* (i.e., actively self-harming or unable to move away from means to self-harm, suicidal thoughts with intent and/or a plan, recent suicide attempt), and *abuse, assault, or violence* (i.e., current/ongoing abuse, recent assault, recent domestic violence). High-risk contacts are the most likely to require immediate intervention from the crisis line volunteer, including active rescue.

### Positive/helpful Experiences

The one-item positive experience rating question had a restricted range with volunteers only endorsing the high end (3 = *Very positive* and 4 = *Extremely positive*) of the 5-point scale; the weighted average across all volunteers and all time points was high: *M* = 3.65 (*SD* = 0.35). All volunteers endorsed some positive aspects of working on the crisis line. The most positive/helpful aspects of working on the t2t crisis line, endorsed by all volunteers, were *helping others*, *skills learned*, and *work is a good match with my skills and values* (see Table 3a for a full list). In terms of positive impact on their lives outside of the crisis line, all volunteers reported *greater empathy for others*, and *improved listening and communication skills* (see Table 4a for a full list).

### Negative/unhelpful Experiences

Across all surveys completed, psychological distress was in the moderate range (*M* = 11.82, *SD* = 2.41) and secondary traumatic stress was in the low range (*M* = 18.64, *SD* = 3.28). The one-item negative experience rating question had a restricted range with volunteers only endorsing the low end (0 = *Not at all negative* and 1 = *A little negative*) of the 5-point scale; the weighted average across all volunteers and all time points was low: *M* = 0.49 (*SD* = 0.35).

The most negative/unhelpful aspects of the crisis line work endorsed by volunteers were: *stressful work* (70%), *negative impact on my mental health* (40%), and *minimizing my own problems—compared to those who use the crisis line* (25%; see Table 3b). In addition to the response options provided, volunteers wrote in some additional negative/unhelpful aspects of the crisis line work that were not accounted for by the existing categories. Specifically, 20% reported that there were some interpersonal stressors within the crisis line community (either with volunteers or supervisors), and 10% reported that they did not feel competent or efficacious. It is important to note that concerns about competency and efficacy were reported in baseline surveys but not during follow-up surveys—potentially indicating that this was a concern earlier in their training but less so after they gained more experience. Further, in terms of negative/unhelpful impact on their lives outside of the crisis line, volunteers reported: *increased pressure to support others’ mental health because of work on the crisis line* (65%), *emotionally drained* (60%), and *increased stress or anxiety* (40%; see Table 4b). Of note, 10% of the sample did not report any negative experiences–either on the crisis line or on their lives outside of the crisis line.

## Discussion

This pilot study is the first to examine the experience of teen-to-teen (t2t) crisis line volunteers. Given the mental health crisis among youth and the national recognition of crisis lines as a critical component of suicide prevention and mental health services, research related to the variety of crisis line services for youth is more relevant than ever. There are four main findings of this pilot study.

First, this study identified that youth volunteers have a range of motivations for pursuing t2t crisis line work. Consistent with research in adults, the most common reasons for youth were to help others (Aguirre & Bolton, 2013; Hector & Aguirre, 2009; Mishara & Giroux, 1993; Praetorius & Machtmes, 2005; Sundram et al., 2018) and to gain new skills (Mishara & Giroux, 1993; Praetorius & Machtmes, 2005; Sundram et al., 2018). Another common motivation for joining the crisis line was because of their own or close others’ lived experience, consistent with prior qualitative research in adults (Aguirre & Bolton, 2013), and adults’ motivation to increase self-knowledge through crisis line work (Praetorius & Machtmes, 2005; Rath, 2008). Related to lived experience, more than half the sample reported a history of suicidal thoughts and behaviors before joining the crisis line. Additional research is needed to understand the safety of this work for youth over time, and particularly for youth with lived experience who may be more vulnerable to the negative impact of this work. Beyond motivations identified in prior work with adults, several volunteers reported that they wanted to destigmatize mental health conditions, and to address the lack of accessible mental healthcare for youth. These motivations are consistent with research indicating that some aspects of mental health stigma are decreasing with younger cohorts (Pescosolido et al., 2021).

Second, this study found that youth volunteers respond to a range of contacts (across texts, chats, calls, emails) at the t2t crisis line. The most common contacts, reported by all volunteers, involved mental health symptoms and relationship difficulties. Other common contacts included academic stress, bullying, and COVID-19 stress. Moreover, most youth volunteers reported responding to high-risk contacts related to suicide/self-injury or abuse/assault/violence. These contacts are most likely to need immediate intervention, such as active rescue or emergency services, and can be the most stressful. It is important to note that the content of contacts responded to by t2t crisis line youth volunteers are similar to those reported by adult crisis line responders (Ramchand et al., 2017).

Third, t2t volunteers identified many positive experiences on the t2t crisis line. Volunteers reported overall high positive experiences (on a one-item scale). In addition, volunteers reported a range of positive experiences on the crisis line (e.g., helping others and learning new skills), which is in line with some of the most common motivations for pursuing this work (Aguirre & Bolton, 2013; Hector & Aguirre, 2009; Mishara & Giroux, 1993; Praetorius & Machtmes, 2005; Sundram et al., 2018). In addition, volunteers identified the positive impact this work had on their life outside of the crisis line, including greater empathy for others (Paterson, 2009) and improved listening and communication skills. Although limited prior research has examined the positive experiences of crisis line responders, the current findings align with studies in adults finding that crisis line responders report high satisfaction with the work (Hellman & House, 2006) and feelings of personal accomplishment (Roche & Ogden, 2017). In sum, this pilot study provides preliminary evidence that the positive experiences gained through this work may go beyond the crisis line experience.

Fourth and finally, this pilot study provides preliminary evidence of some of the negative aspects of this crisis line work for youth. Volunteers reported overall low negative experiences (on a one-item scale), as well as low levels of secondary traumatic stress. Although preliminary, these findings do align with previous research with adults, which did not find increases in secondary traumatic stress among crisis line responders (Dunkley & Whelan, 2006; Furlonger & Taylor, 2013; Kitchingman et al., 2018a). However, the t2t volunteers also identified that this work can be stressful and, in some cases, have a negative impact on their mental health. The long-term effects of this work on their mental health and well-being are important areas for future research. In addition, volunteers identified the negative/unhelpful impact that this work had on their life outside of the crisis line, including feeling emotionally drained and increased pressure to support others’ mental health because they are trained as a crisis line responder. It is important to note that this pilot data was collected during the COVID-19 pandemic, as well as during some significant regional stressors for the youth working on these crisis lines (e.g., wildfires in Oregon). It is unclear how negative experiences (e.g., feeling emotionally drained) may have been impacted by these significant life stressors experienced outside of the crisis line.

There are limitations of this pilot study that warrant discussion. First, the current sample was small and primarily identified as cisgender female and White, which is not representative of the population of t2t crisis line volunteers, particularly in terms of racial diversity. Future studies should employ strategies specifically aimed at recruiting a more representative sample of the diverse population of youth volunteers. For instance, this pilot study did not provide any monetary benefit, which will be important for future studies to compensate volunteers for the effort and expertise they are providing. In addition, a variety of recruitment strategies (not just flyers, but informational sessions) could be employed to address questions and concerns that youth may have about participating in a research study (Knight et al., 2009). Second, because the sample was small and attrition was high, the study was limited in its ability to examine the impact of crisis line volunteerism over time–an important direction for future research. Future research would benefit from recruiting volunteers shortly after training (when they have a one-year commitment and are less likely to drop out of the study) and providing monetary compensation (for the effort and expertise they are providing). Third, this study assessed the content of crisis line contacts but not the modes of communication (e.g., differences in calls vs. texts), which may be important for understanding volunteers’ experiences and thus an important direction for future research. Finally, this pilot study was exploratory and the findings are mainly descriptive about youth volunteers’ motivations for pursuing crisis line work, and their positive and negative experiences while engaging in this work. Building on these preliminary findings, the next step in this program of research is to test the impact, and critically the safety, of t2t crisis lines for youth volunteers.

## Conclusions

This pilot study is the first examination of t2t crisis line volunteers in collaboration with two of the largest t2t lines in the U.S. Findings provide preliminary information about the positive and negative experiences of adolescents engaging in crisis line work. Future research with t2t crisis lines will inform the field about how this crisis resource may help vulnerable youth and its role in the larger suicide prevention landscape.

## Electronic Supplementary Material

Below is the link to the electronic supplementary material.

Supplementary Material 1

## Data Availability

The data that support the findings of this study are available from the corresponding author upon reasonable request.

## Footnotes

1 Teen Line, previously a standalone organization, became part of Didi Hirsch in July 2022 and is now called Teen Line, a Program of Didi Hirsch.
