# Supplementary material for: Evaluating the Experience of Teen-to-Teen Crisis Line Volunteers: A Pilot Study
Source: Community Ment Health J. 2024 Jun 4;60(7):1422–33. doi: 10.1007/s10597-024-01298-z (PMC11408403; doi:10.1007/s10597-024-01298-z)
Supplement: Supplementary file 1 — Supplementary Material 1 [file 10597_2024_1298_MOESM1_ESM.pdf]

Supplement 1

Codebook for analyzing responses about motivations for joining the teen-to-teen crisis lines.

**Original Categories (created based on previous research with adults)**		
**Code**	**Definition**	**Example of Construct** ***(not a direct quote)***
Help others and give back to the community	Volunteer describes wanting to provide resources to their peers and/or community more broadly.	I wanted to help other teens.
Given my own or close others lived experience with mental health issues *[based on 'personal loss' category found in previous research with adults]*	Volunteer describes their own lived experience, or someone else's lived experience with specific mental health conditions or symptoms.	There are several people in my family with mental health issues.
Gain new skills	Volunteer describes gaining skills that are focused on personal development or helping others.	I wanted to learn the tools to help others in crisis.
**New Categories (identified during the coding process)**		
**Code**	**Definition**	**Example of Construct (not a direct quote)**
Learn more about mental health	Volunteer describes wanting to learn more about mental health in general (but not specific to their lived experience).	I wanted to learn more about the mental health issues teens my age face.
Destigmatize or raise awareness about mental health conditions	Volunteer describes mental health stigma in their community or wanting to destigmatize mental health.	Growing up there was a stigma about people with mental health conditions.
Respond to demand for mental health services and need for equitable mental healthcare	Volunteer describes the need for mental health resources in their community or wanting to help make mental health resources more available to vulnerable groups.	Some people don't have access to the mental health services they need. I wanted to help fill that gap.
